# Supplementary material for: Comparative multiomics analysis of cell physiological state after culture in a basket bioreactor
Source: Sci Rep. 2022 Nov 23;12:20161. doi: 10.1038/s41598-022-24687-4 (PMC9686226; doi:10.1038/s41598-022-24687-4)
Supplement: Supplementary file 1 — Supplementary Information 1. [file 41598_2022_24687_MOESM1_ESM.zip › raw data/Transcriptomic raw data/6.Enrichment/1.GO/TE1vsTC1/GSEA/TE1/CELLULAR_RESPONSE_TO_ORGANIC_CYCLIC_COMPOUND/CELLULAR_RESPONSE_TO_ORGANIC_CYCLIC_COMPOUND(GO_0071407).html]

Details for gene set CELLULAR\_RESPONSE\_TO\_ORGANIC\_CYCLIC\_COMPOUND(GO:0071407)[GSEA]


|  || Dataset | readcount.sample |
| Phenotype | sample.cls |
| Upregulated in class | TE1 |
| GeneSet | CELLULAR\_RESPONSE\_TO\_ORGANIC\_CYCLIC\_COMPOUND(GO:0071407) |
| Enrichment Score (ES) | 0.5021175 |
| Normalized Enrichment Score (NES) | 2.0703816 |
| Nominal p-value | 0.0 |
| FDR q-value | 0.17100555 |
| FWER p-Value | 0.32 |
Table: GSEA Results Summary

  

Fig 1: Enrichment plot: CELLULAR\_RESPONSE\_TO\_ORGANIC\_CYCLIC\_COMPOUND(GO:0071407)
  
Profile of the Running ES Score & Positions of GeneSet Members on the Rank Ordered List

  

| PROBE | DESCRIPTION   (from dataset) | GENE SYMBOL | GENE\_TITLE | RANK IN GENE LIST | RANK METRIC SCORE | RUNNING ES | CORE ENRICHMENT || 1 | 103219594 | RORB |  |  | 647 | 8.838 | 0.0795 | Yes |
| 2 | 103229160 | ESR2 |  |  | 655 | 8.298 | 0.1810 | Yes |
| 3 | 103219133 | NR4A3 |  |  | 674 | 7.430 | 0.2714 | Yes |
| 4 | 103240625 | NR2E1 |  |  | 693 | 6.990 | 0.3563 | Yes |
| 5 | 103238335 | NR4A1 |  |  | 821 | 4.959 | 0.4115 | Yes |
| 6 | 103236990 | HNF4G |  |  | 1239 | 2.963 | 0.4292 | Yes |
| 7 | 119619039 | LOC119619039 |  |  | 1281 | 2.852 | 0.4624 | Yes |
| 8 | 103245382 | NR2E3 |  |  | 1377 | 2.643 | 0.4905 | Yes |
| 9 | 103217123 | NR4A2 |  |  | 1792 | 2.244 | 0.4995 | Yes |
| 10 | 119626588 | LOC119626588 |  |  | 2741 | 1.781 | 0.4790 | Yes |
| 11 | 103241764 | NR1D2 |  |  | 2952 | 1.718 | 0.4907 | Yes |
| 12 | 103243522 | NR1D1 |  |  | 3324 | 1.640 | 0.4942 | Yes |
| 13 | 103238386 | RARG |  |  | 4464 | 1.465 | 0.4612 | Yes |
| 14 | 103244720 | NR3C1 |  |  | 4562 | 1.452 | 0.4747 | Yes |
| 15 | 103238886 | NR2C1 |  |  | 4956 | 1.407 | 0.4744 | Yes |
| 16 | 103228027 | PPARG |  |  | 4981 | 1.403 | 0.4905 | Yes |
| 17 | 103228057 | NR2C2 |  |  | 5509 | 1.348 | 0.4835 | Yes |
| 18 | 103248320 | PGR |  |  | 5838 | 1.319 | 0.4850 | Yes |
| 19 | 103230089 | ESRRG |  |  | 5947 | 1.306 | 0.4962 | Yes |
| 20 | 103241767 | THRB |  |  | 6169 | 1.286 | 0.5021 | Yes |
| 21 | 103224681 | NR2F1 |  |  | 6748 | 1.240 | 0.4915 | No |
| 22 | 103239886 | NR6A1 |  |  | 8603 | 1.111 | 0.4222 | No |
| 23 | 103221675 | RXRB |  |  | 9515 | 1.053 | 0.3944 | No |
| 24 | 103236358 | NR3C2 |  |  | 9773 | 1.037 | 0.3956 | No |
| 25 | 103235078 | NR1H2 |  |  | 9978 | 1.022 | 0.3990 | No |
| 26 | 103231245 | NR2F2 |  |  | 10001 | 1.021 | 0.4105 | No |
| 27 | 119627299 | PPARA |  |  | 10362 | 1.000 | 0.4067 | No |
| 28 | 103243521 | THRA |  |  | 10746 | 0.973 | 0.4015 | No |
| 29 | 103221615 | PPARD |  |  | 10998 | 0.957 | 0.4020 | No |
| 30 | 103234692 | LOC103234692 |  |  | 11468 | 0.928 | 0.3924 | No |
| 31 | 103245526 | RORA |  |  | 11632 | 0.917 | 0.3964 | No |
| 32 | 103243515 | RARA |  |  | 12086 | 0.889 | 0.3871 | No |
| 33 | 103234125 | NR2F6 |  |  | 12312 | 0.875 | 0.3877 | No |
| 34 | 103238936 | NR1H4 |  |  | 14828 | 0.719 | 0.2841 | No |
| 35 | 103239669 | RXRA |  |  | 15149 | 0.698 | 0.2783 | No |
| 36 | 103233248 | ESRRA |  |  | 15193 | 0.695 | 0.2849 | No |
| 37 | 103236235 | NR1H3 |  |  | 15708 | 0.661 | 0.2700 | No |
| 38 | 103223683 | TOMM40L |  |  | 15832 | 0.652 | 0.2725 | No |
| 39 | 103238213 | VDR |  |  | 16850 | 0.580 | 0.2342 | No |
| 40 | 103241772 | RARB |  |  | 17261 | 0.553 | 0.2226 | No |
| 41 | 103224018 | RORC |  |  | 19847 | 0.296 | 0.1106 | No |
| 42 | 103240732 | ESR1 |  |  | 19909 | 0.285 | 0.1114 | No |
| 43 | 103229373 | ESRRB |  |  | 21107 | 0.000 | 0.0579 | No |
| 44 | 103239887 | NR5A1 |  |  | 21275 | 0.000 | 0.0504 | No |
Table: GSEA details
[plain text format]

  

Fig 2: CELLULAR\_RESPONSE\_TO\_ORGANIC\_CYCLIC\_COMPOUND(GO:0071407)
  
Blue-Pink O' Gram in the Space of the Analyzed GeneSet

  

Fig 3: CELLULAR\_RESPONSE\_TO\_ORGANIC\_CYCLIC\_COMPOUND(GO:0071407): Random ES distribution
  
Gene set null distribution of ES for
 **CELLULAR\_RESPONSE\_TO\_ORGANIC\_CYCLIC\_COMPOUND(GO:0071407)**
